# Left bundle branch area pacing vs. biventricular pacing significantly improves clinical outcomes and cardiac remodeling in cardiac resynchronization therapy: a systematic review and meta-analysis

**DOI:** 10.3389/fcvm.2025.1644033

**Published:** 2025-11-21

**Authors:** Zaixing Zheng, Longfu Jiang, Yi Gao, Xinhui Peng, Haiming Feng, Jinmei Lu

**Affiliations:** 1 Department of Cardiology, Ningbo NO.2 Hospital, Ningbo, Zhejiang, China; 2 Department of Critical Care Medicine, Ningbo Medical Center Li Huili Hospital, Ningbo, Zhejiang, China

**Keywords:** left bundle branch area pacing, biventricular pacing, cardiac resynchronization therapy, heart failure, meta-analysis

## Abstract

**Background:**

Biventricular pacing (BiVP) is the conventional approach for cardiac resynchronization therapy (CRT), yet approximately one-third of patients show no clinical response. Left bundle branch area pacing (LBBAP) enables more physiological ventricular activation through His-Purkinje conduction, but its impact on key clinical endpoints such as all-cause mortality and heart failure hospitalization (HFH) remains debated.

**Methods:**

A systematic search of PubMed, Embase, Cochrane Library, and CNKI (to May 3, 2025) identified 24 studies encompassing 6,538 patients. Study quality was assessed using Cochrane RoB 2.0 and the Newcastle–Ottawa Scale. Subgroup analyses (by follow-up duration, study design, and sex), leave-one-out sensitivity analysis, and meta-regression were performed to assess result robustness and heterogeneity sources. Trim-and-fill correction was applied to adjust for potential publication bias.

**Results:**

LBBAP was associated with a markedly lower risk compared to BiVP across several clinical outcomes. Specifically, it significantly reduced the risk of the composite endpoint (HR: 0.67, 95% CI: 0.59–0.75), all-cause mortality (HR: 0.83, 95% CI: 0.71–0.96), and HFH (HR: 0.58, 95% CI: 0.50–0.67). Echocardiographic outcomes further supported LBBAP superiority, with higher rates of echocardiographic response (OR: 1.57, 95% CI: 1.36–1.81) and super-response (OR: 2.12, 95% CI: 1.62–2.76). Improvements in left ventricular ejection fraction (LVEF) were greater with LBBAP at both 3–6 months (MD: 5.31%, 95% CI: 4.63–5.99) and ≥12 months (MD: 4.43%, 95% CI: 2.27–6.60). Similarly, left ventricular end-diastolic diameter (LVEDD) reductions were more pronounced at 3–6 months (MD: −3.48 mm, 95% CI: −5.76 to −1.21) and ≥12 months (MD: −2.86 mm, 95% CI: −5.05 to −0.68).

**Conclusions:**

These findings indicate that LBBAP provides superior clinical and structural outcomes compared to BiVP in patients undergoing CRT. Large-scale, multicenter randomized controlled trials are warranted to confirm these results, assess long-term efficacy, and elucidate gender-specific variations to optimize evidence-based CRT delivery.

**Systematic Review Registration:**

https://www.crd.york.ac.uk/prospero/display_record.php?ID=CRD420251055488, PROSPERO CRD420251055488.

## 1 Introduction

Cardiac resynchronization therapy (CRT) is a key intervention for improving ventricular synchrony in patients with heart failure (HF) and reduced left ventricular ejection fraction (LVEF), particularly those with left bundle branch block (LBBB) or an anticipated right ventricular pacing burden exceeding 40% (1). Biventricular pacing (BiVP), the conventional CRT approach, has been shown to enhance functional capacity and reduce all-cause mortality by synchronizing ventricular activation through an endocardial right ventricular lead and an epicardial left ventricular lead positioned via the coronary sinus (2, 3). However, approximately one-third of patients fail to respond to BiVP in clinical practice (4), possibly due to non-physiological activation sequences that induce secondary electromechanical dyssynchrony (5). Mechanistically, chronic epicardial left ventricular pacing may produce less physiological activation and potentially promote adverse ventricular remodeling over time compared with conduction system pacing (6). This hypothesis, however, requires confirmation through direct long-term comparative studies.

Left bundle branch area pacing (LBBAP) has emerged as a physiological pacing strategy that achieves ventricular resynchronization through direct activation of the His–Purkinje network. By circumventing the non-physiological myocardial stimulation inherent to epicardial pacing, LBBAP represents a promising alternative to conventional BiVP (7). Several studies have shown that LBBAP yields higher procedural success rates than BiVP (8) and more effectively narrows QRS duration and improves ventricular remodeling parameters (9). Moreover, LBBAP is often associated with shorter procedural and fluoroscopy times (10, 11). Nevertheless, the evidence remains heterogeneous; certain registries report no significant procedural advantage, likely reflecting variations in operator experience and the procedural learning curve. Although LBBAP demonstrates superior electrophysiological and remodeling outcomes, evidence regarding hard clinical endpoints such as all-cause mortality and heart failure hospitalization (HFH) remains inconsistent. Parlavecchio et al. reported a ∼40% reduction in HFH with LBBAP compared with BiVP, though no difference in mortality was observed (9). Similarly, Jin et al. found that LBBAP reduced HFH but did not significantly affect all-cause mortality (11). In contrast, Leventopoulos et al. (12) reported reductions in both mortality and HFH with LBBAP; however, these results were largely driven by the study from Vijayaraman et al. (13), as significance was lost after its exclusion. Such discrepancies likely stem from limited follow-up durations and small sample sizes.

Recently, large multicenter studies have provided more robust comparative data. Morcos et al. (14) analyzed outcomes from 2,579 patients and reported significantly lower risks of the composite endpoint and HFH in the LBBAP group compared with BiVP. In a cohort of 539 patients, Tedrow et al. (15) found that LBBAP significantly reduced the primary endpoint in male patients but not in females. Conversely, Subzposh et al. (16) demonstrated that female patients receiving LBBAP experienced significant reductions in both the composite endpoint and HFH. Similarly, Vijayaraman et al. (13) observed lower composite event rates with LBBAP in a 1,778-patient cohort. Collectively, these findings highlight the growing evidence base for LBBAP across diverse patient subgroups and reinforce its potential as a superior CRT modality.

The present study aims to systematically compare LBBAP and BiVP for CRT in patients with HF through a comprehensive systematic review and meta-analysis. We evaluated group differences in composite clinical endpoints, all-cause mortality, HFH, and echocardiographic outcomes to provide evidence-based guidance for clinical practice and future research.

## 2 Methods

### 2.1 Research registration and reporting standards

This meta-analysis adhered to the Preferred Reporting Items for Systematic Reviews and Meta-Analyses (PRISMA) 2020 guidelines (17) and was registered on PROSPERO (registration number: CRD420251055488). Because only published aggregate data were used, Institutional Review Board approval was not required.

### 2.2 Search strategy

Comprehensive searches were conducted across PubMed, Embase, the Cochrane Library, and CNKI from inception to May 3, 2025. The strategy combined Medical Subject Headings and free-text terms (e.g., “Cardiac Resynchronization Therapy,” “left bundle branch area pacing”) using structured Boolean operators (“AND”/ “OR”). Reference lists of included studies were manually screened to identify additional eligible publications. No language restrictions were applied, while case reports, reviews, editorials, and commentaries were excluded based on study design. The search was limited to major electronic databases primarily indexing peer-reviewed articles; thus, grey literature sources (e.g., preprint servers, conference proceedings) were not systematically searched. The detailed search protocol is provided in Supplementary Table S1.

### 2.3 Inclusion criteria and study selection

Inclusion Criteria: (1) Population: Adults (≥18 years) with heart failure with reduced ejection fraction (HFrEF) who met current guideline-based indications for cardiac resynchronization therapy (CRT). (2) Intervention: Controlled comparative studies directly evaluating left bundle branch area pacing (LBBAP) vs. biventricular pacing (BiVP). (3) Outcomes: Studies reporting at least one of the following: composite clinical endpoints, all-cause mortality, heart failure hospitalization (HFH), or echocardiographic parameters (LVEF, LVEDD). The primary composite endpoint for this analysis was predefined as a combination of all-cause mortality and HFH. Studies reporting expanded composite endpoints (e.g., including cardiac transplant, left ventricular assist device implantation, or recurrent HFH) were also included; however, the specific composition of each study's endpoint was explicitly documented in the data extraction process and considered during the analysis.

Exclusion Criteria: (1) Study Design and Data: Non-comparative studies; studies with combined pacing interventions lacking separate analyses (e.g., LBBAP and His-bundle pacing reported jointly); or studies with incomplete datasets. (2) Population and Intervention: Non-adult or non-HF populations; CRT studies involving LBBAP used solely for optimization or as an adjunct; right ventricular pacing or other non-CRT interventions; or studies without clearly defined LBBAP implantation success criteria. (3) Publication Characteristics: Follow-up duration <3 months; total sample size <10 patients; single-arm designs; or publications limited to case reports, reviews, editorials, or conference abstracts.

The literature screening process followed the PRISMA 2020 guidelines. All retrieved records were imported into EndNote X8 for duplicate removal. The remaining studies underwent independent title and abstract screening by two reviewers (ZZX and LJM) according to the predefined eligibility criteria. Discrepancies were resolved by discussion; unresolved cases were adjudicated by a senior reviewer (JLF). Full-text assessment of potentially eligible studies was performed by the same reviewers using identical criteria. Final inclusion for meta-analysis was determined through consensus.

### 2.4 Data extraction

Data were independently extracted by two reviewers (ZZX and LJM) using a standardized Excel template, capturing: (1) Study characteristics: Author(s), publication year, country, number of study centers, and study design. (2) Patient demographics: Total enrollment, sample size per group, mean age, female proportion, prevalence of ischemic cardiomyopathy (ICM), and pacing indications. (3) Intervention details: Pacing modality (LBBAP vs. BiVP) and follow-up duration. For this meta-analysis, “LBBAP” encompassed both left bundle branch pacing (LBBP) and left ventricular septal pacing (LVSP) as reported in the included studies. (4) Outcomes: Composite clinical endpoints, all-cause mortality, HFH, and echocardiographic measures (LVEF and LVEDD). All discrepancies were resolved by consensus; unresolved disagreements were reviewed by a senior third investigator (LJF). Extracted data were cross-verified before being imported into statistical software for analysis.

### 2.5 Quality assessment

Randomized controlled trials (RCTs) were evaluated using the Cochrane Risk of Bias 2.0 tool (18), while observational studies were assessed with the Newcastle–Ottawa Scale (NOS) (19). Two reviewers (ZZX and LJM) conducted assessments independently, resolving disagreements through discussion or third-party adjudication (JLF) to ensure methodological rigor and objectivity.

### 2.6 Statistical analysis

All statistical analyses were performed using Stata 18.0. Time-to-event outcomes (composite endpoints, all-cause mortality, and HFH) were pooled as hazard ratios (HRs) with 95% confidence intervals (CIs) using the generic inverse-variance method on log-HRs, including only adjusted estimates. Dichotomous outcomes (echocardiographic response and super-response rates) were synthesized as odds ratios (ORs) via the Mantel–Haenszel method, while continuous variables (LVEF and LVEDD improvements) were analyzed as mean differences (MDs) using inverse-variance weighting. When studies reported medians and interquartile ranges (IQRs), means were approximated from medians, and standard deviations were estimated as IQR/1.35. Between-study heterogeneity was assessed using Cochran's *Q* and *I*^2^ statistics. A fixed-effect model was applied when *I*^2^ < 50% and *P* ≥ 0.1; otherwise, a random-effects model with Knapp–Hartung correction was used. For highly heterogeneous outcomes (*I*^2^ ≥ 50%), meta-regression with restricted maximum likelihood was conducted to explore potential sources of variation, including differences in sex, NICM prevalence, LBBB proportion, QRS duration, and follow-up length. Subgroup analyses were stratified by follow-up duration (≤1 year vs. >1 year), study design, and sex. Sensitivity analyses included a leave-one-out approach and trim-and-fill correction for publication bias. Bias was assessed using the Harbord test for dichotomous outcomes and the Egger test for continuous outcomes, complemented by funnel plots. All tests were two-tailed, with statistical significance defined as *P* < 0.05.

## 3 Results

### 3.1 Study selection

**Figure 1:**
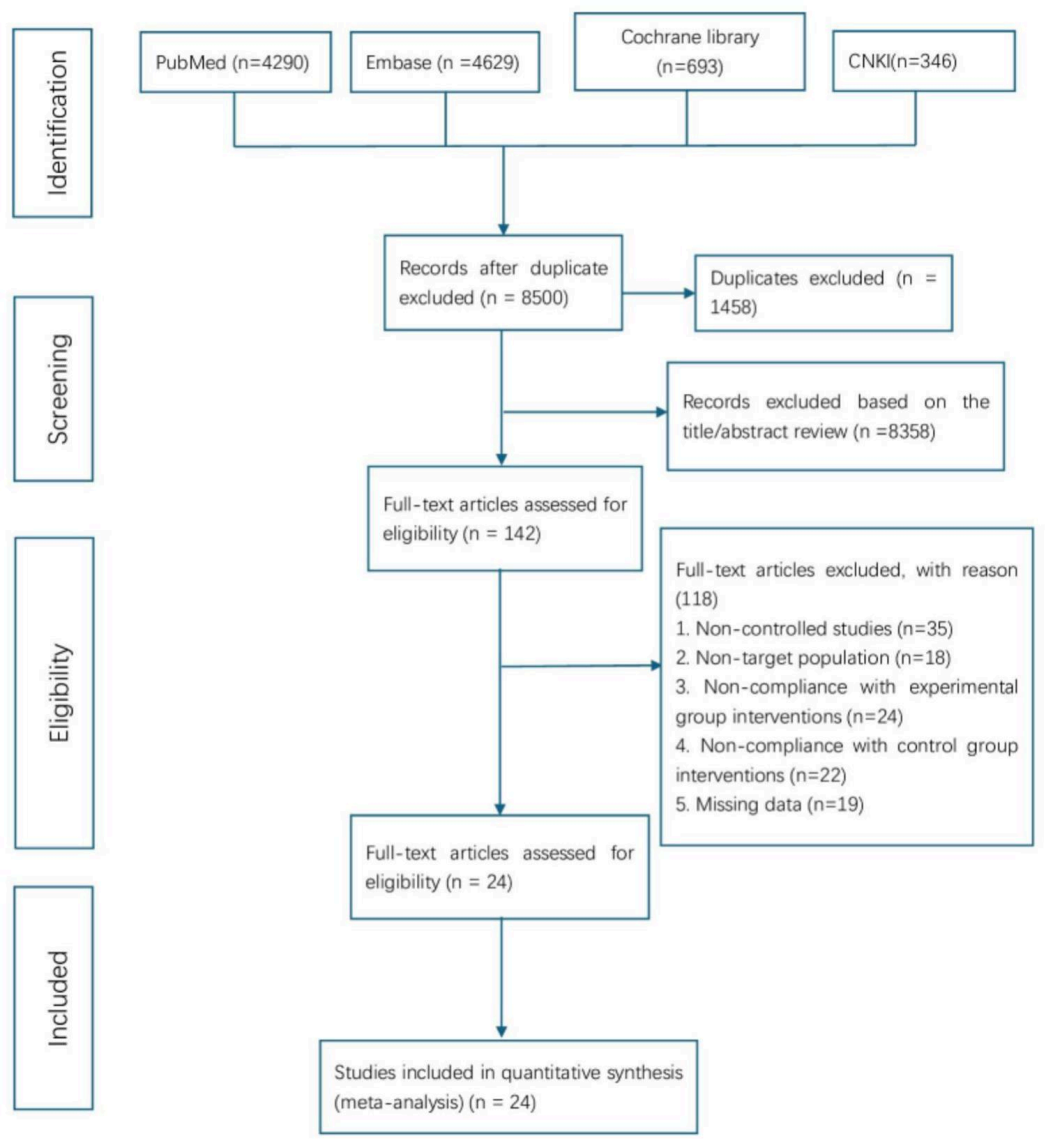
The PRISMA flow chart of literature screening and selection process.

The initial database search yielded 9,958 records. After importing into EndNote X8 for duplicate removal, 1,458 records were excluded, leaving 8,500 unique studies. Title and abstract screening eliminated 8,358 irrelevant papers, and 142 articles underwent full-text review. Of these, 118 were excluded for failing to meet inclusion criteria—most commonly due to non-controlled design, non-target populations, inconsistent interventions, or incomplete data. Ultimately, 24 studies met the eligibility criteria and were included in the meta-analysis. The study selection process conformed to PRISMA guidelines, with detailed steps illustrated in Figure 1.

### 3.2 Characteristics of the included studies

A total of 24 studies were included, comprising one randomized controlled trial (20) and 23 observational studies (13–16, 21–39). Of these, 17 were prospective and 7 retrospective, published between 2020 and 2025. The studies originated primarily from China, the United States, the Netherlands, Australia, and Colombia, and included 9 single-center and 15 multicenter designs. The median follow-up duration was 13.8 months (range 4–37 months). The pooled cohort comprised 6,538 patients (LBBAP *n* = 2,829; BiVP *n* = 3,709). The LBBAP group included a higher proportion of women (35.8% vs. 30.9%; *P* < 0.05) and a higher prevalence of hypertension (63.5% vs. 61.0%; *P* < 0.05), but lower proportions of ischemic HF (29.9% vs. 31.9%; *P* < 0.05) and LBBB (64.3% vs. 68.5%; *P* < 0.05). Baseline LVEDD was smaller in the LBBAP group (62.2 ± 9.0 mm vs. 64.5 ± 9.2 mm; *P* < 0.05), whereas the baseline QRS duration was wider (162.4 ± 26.7 ms vs. 161.2 ± 23.4 ms; *P* < 0.05). No significant differences were observed in age, diabetes, atrial fibrillation, baseline LVEF, or medication profiles. As Subzposh et al. (2024) and Tedrow et al. (2024) reported sex-stratified data, male and female subsets were designated Subzposh 2024_a, Subzposh 2024_b, Tedrow 2024_a, and Tedrow 2024_b. Moreover, since Subzposh et al. (2024) and Vijayaraman et al. (2023) analyzed overlapping patient cohorts, the Subzposh 2024 dataset was used exclusively for sex-based subgroup analyses, whereas Vijayaraman 2023 data were applied to all other analyses. To eliminate any ambiguity, we explicitly confirm that Subzposh 2024 and Vijayaraman 2023 data were never combined in the same model for any endpoint. This rigorous separation ensures that no overlapping patient cohorts were included in pooled effect estimates. Comprehensive study characteristics are summarized in Tables 1, 2.

**Table 1:** General characteristics of the included studies.

ID	Authors	Year	Country	Centers	Design	Follow-up, month	Experimental group	Simplified pacing indications	NOS/RoB 2.0	Quality assessment
1	Morcos, R.	2025	12 countries	18	Retrospective study	34 ± 15	LBBAP. Success if V1 shows Qr/qR, with left bundle potential, etc. Post—op: optimize AV delay, maximize LV—RV offset (80–100 ms) or program to LV—only pacing	NYHAII–IV, LVEF ≤ 50%, CRT-eligible or ventricular pacing > 40%	9	high quality
2	Zhu, H.	2024	China	2	Prospective study	28.8 ± 15.8	Group 1: LBBP (3,830 lead + C315 sheath, advanced to left septum, confirmed by left bundle capture). Optimize AV delay. Group 2: LVSP (V1 Qr/qR/QS, no sudden LVAT shortening). If LBBAP fails shortest QRS, switch to BiVP	HF, LVEF < 50% (including LBBB: QRS ≥ 130 ms, LVEF ≤ 35%)	7	high quality
3	Wang, S.	2024	China	1	Retrospective study	16 (12,30)	LBBAP (success with Qr/qR + one of four criteria). If not, LVSP. For CRTD, LBBP lead connects to LV port, no coronary sinus lead	LBBB, QRS ≥ 130 ms, LVEF ≤ 40%, NYHAII–IV, GDMT ≥ 3m	7	high quality
4	Verstappen, A. A. A.	2024	the Netherlands and the Czech Republic	2	Prospective study	12	LBBAP (3,830 lead + C315HIS sheath, confirmed by pseudo—RBBB, LVAT ≤95 ms, V6–V1 interval >33 ms). Program to bipolar DDD	Sinus rhythm, NYHAII–IV, LVEF ≤ 35%, complete LBBB (male QRS > 140 ms, female > 130 ms)	7.5	high quality
5	Tedrow, U. B.	2024	The United States, Colombia, and Argentina	4	Prospective study	400.5 (207–624) days	LBBAP (C315HIS sheath, 9—zone method, 3,830 lead). Success by current LBB capture criteria	LVEF ≤ 35%+LBBB; or LVEF ≤ 40%+RV pacing > 40%	9	high quality
6	Subzposh, F. A.	2024	10 countries	NR	Retrospective study	25.2 ± 15.6	LBBAP (V1 Qr/qR + conditions like left bundle potential). Success if meeting criteria	NYHAII–IV, EF < 35%, QRS > 130 ms or frequent pacing	8.5	high quality
7	Shroff, J. P.	2024	Australia	1	Prospective study	33.7 ± 10.6	LBBAP (3,830 lead via C315—HIS sheath after right ventricular septum defibrillator lead). Confirmed by specific ECG	HF referral, met CRT criteria after GDMT	8	high quality
8	Liang, Y.	2024	China	2	Retrospective study	28	LBBAP (3,830 lead via C315HIS sheath, confirmed by terminal R in V1, LVAT <90 ms, etc.)	NYHAII–IV, QRS ≥ 130 ms, LVEF ≤ 35%; or high AV block + pacing > 40%+LVEF < 40%	9	high quality
9	Li, J.	2024	China	1	Retrospective study	37 ± 19	LBBAP via left axillary vein (3,830 lead + C315 HIS sheath). Confirmed by V1 QRS morphology, Stim—LVAT. If failed, switch to BiVP	Complete LBBB, LVEF ≤ 35%, NYHAII–IV	8	high quality
10	Diaz, J. C.	2024	Colombia, America and Argentina	5	Prospective study	399days	LBBAP (3,830 lead via C315HIS sheath, confirmed by V1 Qr/qR/rSR0, LVAT <80 ms, etc.). If not, LVSP	Strauss LBBB + LVEF ≤ 35%; or LVEF < 40%+RV pacing > 40%, NYHAII–IV after GDMT	9	high quality
11	Chen, X.	2024	China	4	Prospective study	24	LBBAP (3,830 lead + C315 His sheath, advanced 1–2 cm from His—apex line). Confirmed by RBBB morphology, Sti—LVAT sudden shortening ≤1.5 V/0.5 ms	CRT non-responders (>12 m, post-OMT/optimization), *Δ*LVEF < 5%	7	high quality
12	Vijayaraman, P.	2023	10 countries	15	Retrospective study	33 ± 16	LBBAP (3,830 lead via fixed/deflectable sheath, confirmed by Qr/qR + one condition). Some with coronary sinus LV lead for LOT—CRT	NYHAII–IV, LVEF ≤ 35%, CRT-eligible or pacing > 40%	7	high quality
13	Rademakers, L. M.	2023	The Netherlands	1	Prospective study	6	LBBAP (3,830 lead + C315HIS sheath, confirmed by RBBB morphology, LVAT ≤90 ms). Optional RV backup lead	NYHAII–IV, LVEF ≤ 35%, complete LBBB (male QRS > 140 ms, female > 130 ms)	6.5	moderate quality
14	Pathak, R. K.	2024	Australia	1	Prospective study	33.7 ± 10.6	LBBAP (3,830 lead via C315—HIS sheath, confirmed by RBBB in V1, short R—peak time in V6). Right ventricular septum defibrillator lead first	NYHAII–IV, LVEF ≤ 35% post-OMT; SR/AF + IVCD(QRS ≥ 150 ms) or AV block/Strauss LBBB	8	high quality
15	Diaz, J. C.	2023	America, Colombia and Argentina	5	Prospective study	LBBAP 308 (196–419) days;	LBBAP (3,830 lead via C315HIS sheath, 9—zone method, confirmed by V1 Qr/qR/rSR’, LVAT sudden shortening)	Strauss LBBB + LVEF ≤ 35%; or LVEF < 40%+RV pacing > 40%, NYHAII–IV (first CRT)	8	High quality
						BiVP 378 (209.5–552) days				
16	Wang, Y.	2022	China	2	Prospective, randomized, controlled trial	6	LBBAP (3,830 lead + C315 HIS sheath, confirmed by RBBB in V1, LVAT ≤100 ms). For CRT—D, LBBP lead to LV port; for CRT—P, optional coronary sinus lead	SR, Strauss LBBB(male QRS > 140 ms, female > 130 ms), LVEF ≤ 40%, NYHAII–IV, GDMT ≥ 3m	Some concerns	Some concerns
17	Hua, J.	2022	China	1	Prospective study	23.71 ± 4.44	LBBAP (3,830 lead + C315 His sheath, confirmed by RBBB/normal—like QRS, impedance ≥500*Ω*)	QRS > 150 ms, CLBBB, NYHAII–IV, optimized therapy ≥ 3m	6	moderate quality
18	Chen, X.	2022	China	4	Prospective study	12	LBBAP (3,830 lead + C315His sheath, advanced 1 cm from His—apex line). Connected to different ports per device type. Optimize AV delay	Symptomatic HF(NYHAIII–IV), LVEF ≤ 35% post-OMT; SR, QRS ≥ 150 ms, Strauss LBBB	7.5	high quality
19	Zu, L.	2021	China	3	Retrospective study	12	LBBAP (3,830 lead + C315/C314 sheath, confirmed by RBBB morphology)	Dilated CM, CRT-eligible(QRS > 150 ms + LBBB, persistent HF post-therapy, no ischemia in 1 years)	6	moderate quality
20	Wu, Shengjie	2021	China	1	Prospective study	12	LBBAP (confirmed by terminal R in V1, impedance increase). Adjust connection and parameters post—op	Strauss complete LBBB; symptomatic HF, LVEF ≤ 40%	7	high quality
21	Liu, W.	2021	China	3	Prospective study	4.0 ± 1.4	LBBAP (3,830 lead + C315His sheath, confirmed by QRS morphology, LVAT). If LBBAP corrects LBBB or QRS ≤140 ms, use alone; else sequential pacing	Symptomatic, LVEF ≤ 35% post-GDMT ≥ 3 m; CLBBB, QRS ≥ 130 ms	6.5	moderate quality
22	Wang, Y.	2020	China	1	Prospective study	6	LBBAP (3,830 lead + C315 HIS sheath, confirmed by QR/rSR morphology, short LVAT). Program to DDD, optimize AV	Sinus rhythm, LBBB(male QRS > 140 ms, female > 130 ms), LVEF ≤ 35%, NYHAII–IV	7	high quality
23	Li, X.	2020	China	3	Prospective study	6	LBBAP (3,830 lead + C315 His sheath, confirmed by V1 QRS <145 ms/“W” morphology). Adjust lead position by LVAT	HF symptoms, LVEF ≤ 35%+LBBB, GDMT ≥ 4m	7.5	high quality
24	Guo, J.	2020	China	1	Prospective study	14.3 ± 7.2	LBBAP (3,830 lead + C315 HIS sheath, confirmed by V1 “QR/Qr”, short LVAT, threshold <1.5V@0.4 ms). Connect per rhythm	Strauss LBBB, LVEF ≤ 35%, NYHAII–IV	7	high quality

LBBAP, left bundle branch area pacing; NYHA, New York Heart Association; HF, heart failure; LVEF, left ventricular ejection fraction; BiVP, biventricular pacing; LBBB, left bundle branch block; NR, not recorded; CRT, cardiac resynchronization therapy; GDMT, guideline-directed medical therapy; LVAT, left ventricular activation time.

**Table 2:** Baseline characteristic of the included studies.

Baseline characteristic	LBBAP (*n* = 2,829)		Bivp (*N* = 3,709)		*P* value
	Mean ± SD/n (%)	Total, n	Mean ± SD/n (%)	Total, n	
Age, years	68.7 ± 11.6	2,802	68.1 ± 11.3	3,655	>0.05
Female, *n* (%)	1,002 (35.8%)	2,802	1,128 (30.9%)	3,655	<0.05
Hypertension, *n* (%)	1,761 (63.5%)	2,772	2,200 (61.0%)	3,605	<0.05
Diabetes, *n* (%)	1,018 (36.7%)	2,772	1,249 (34.6%)	3,605	>0.05
Atrial fibrillation, *n* (%)	1,010 (36.4%)	2,772	1,343 (37.3%)	3,605	>0.05
Ischemic, *n* (%)	831 (29.9%)	2,781	1,160 (31.9%)	3,635	<0.05
EF, %	28.3 ± 7.2	2,802	28 ± 7.3	3,655	>0.05
LVEDD, mm	62.2 ± 9.0	1,523	64.5 ± 9.2	2,064	<0.05
QRS duration (ms)	162.4 ± 26.7	2,840	161.2 ± 23.4	3,655	<0.05
QRS morphology					
LBBB, *n* (%)	1,528 (64.3%)	2,376	2,025 (68.5%)	2,958	<0.05
Medications					
Beta blockers	2,457 (88.8%)	2,768	3,181 (88%)	3,616	>0.05
ACEI/ARB/ARNI	1,520 (54.5%)	2,789	2,082 (57.3%)	3,636	>0.05
Aldactone antagonists	1,429 (57.3%)	2,494	1,773 (58%)	3,056	>0.05
Diuretics	1,609 (72.7%)	2,213	1,944 (69.8%)	2,784	>0.05

BiVP, biventricular pacing; LBBAP, left bundle branch area pacing; SD, standard deviation; EF, ejection fraction; LVEDD, left ventricular end-diastolic diameter; ACEI, angiotensin-converting enzyme inhibitors; ARB, angiotensin receptor blockers; ARNI, angiotensin receptor neprilysin inhibitors.

### 3.3 Quality assessment and publication bias

The Newcastle–Ottawa Scale (NOS) was used to evaluate 23 observational studies, yielding scores of 6–9 (median: 7.5). Among these, 19 studies (82.6%) were classified as high quality (NOS ≥ 7). The single included randomized controlled trial (RCT), assessed using the Cochrane Risk of Bias 2.0 tool, was rated as “having some concerns” due to insufficient reporting of the randomization process. Detailed quality assessment results are summarized in Table 2. Funnel plot analysis indicated a symmetrical distribution for all-cause mortality (Harbord test, *P* > 0.05). In contrast, significant asymmetry was observed for the composite endpoint, HFH (Harbord test, *P* < 0.05 for both), echocardiographic response rates (Harbord test, *P* < 0.05), and left ventricular (LV) function improvement (Egger's test, *P* < 0.05), suggesting potential publication bias for these outcomes. The trim-and-fill method was employed to adjust for this potential bias. After the imputation of five hypothetical studies for the echocardiographic response rate, the adjusted pooled OR was no longer statistically significant. In contrast, the adjusted results for the composite endpoint, HFH, and LV function improvement remained statistically significant, confirming the robustness of these findings (Supplementary Table S3, Figure 1).

### 3.4 Comparative efficacy analysis of LBBAP vs. BiVP

#### 3.4.1 Composite endpoint

**Figure 2:**
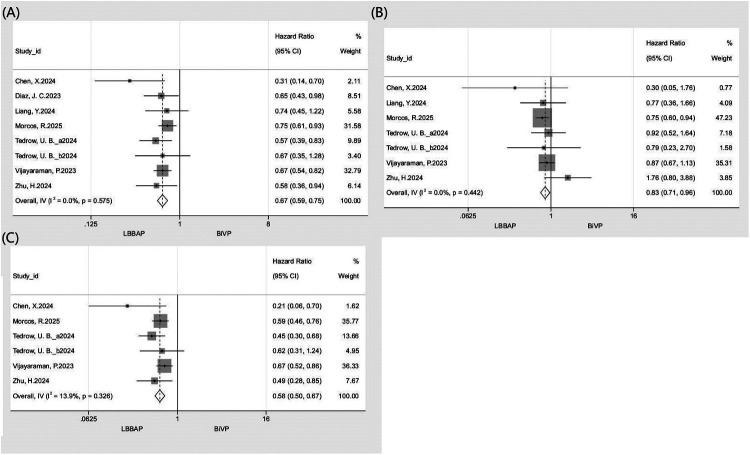
Forest plot comparing clinical outcomes between LBBAP and BiVP. **(A)** All-cause mortality or HFH; **(B)** All-cause mortality; **(C)** HFH.

A pooled analysis of 7 studies (*n* = 5,086) showed that LBBAP was associated with a significantly lower risk of the composite endpoint compared with BiVP (HR: 0.67, 95% CI: 0.59–0.75; *I*^2^ = 0%; Figure 2A). Consistent risk reductions were observed across prespecified subgroups, including prospective (HR: 0.58, 95% CI: 0.47–0.72) and retrospective (HR: 0.71, 95% CI: 0.62–0.82) designs, as well as female (HR: 0.65, 95% CI: 0.46–0.92) and male (HR: 0.79, 95% CI: 0.65–0.95) populations. Sensitivity analysis using the leave-one-out approach demonstrated high result stability (*I*^2^ = 0%; HR range: 0.63–0.68), with no single study exerting a disproportionate influence on the pooled effect. Corresponding forest plots and subgroup data are presented in Figure 2A and Table 3.

**Table 3:** Subgroup analysis of clinical outcomes.

Subgroup	Meta-analysis			Heterogeneity		Test for interaction P
	No. of studies	Total population	HR (95%Cl)	*I*^2^ (%)	*P*	
All-cause mortality or HFH						
Research design						0.12
Prospective study	4	1,257	0.58 (0.47,0.72)	0	0.60	
Retrospective study	3	3,829	0.71 (0.62,0.82)	0	0.74	
Gender						0.34
Female	2	738	0.65 (0.46,0.92)	0	0.91	
Male	2	1,579	0.79 (0.65,0.95)	73	0.05	
All-cause mortality						
Research design						0.44
Prospective study	3	886	1.02 (0.67, 1.56)	24.3	0.27	
Retrospective study	3	3,829	0.80 (0.68, 0.82)	0	0.68	
Gender						0.59
Female	2	739	1.08 (0.66,1.75)	0	0.59	
Male	2	1,579	0.93 (0.73,1.18)	0	0.98	
HFH						
Research design						0.11
Prospective study	3	886	0.47 (0.35, 0.62)	0	0.50	
Retrospective study	2	3,338	0.63 (0.53, 0.75)	0	0.50	
Gender						0.47
Female	2	738	0.47 (0.31,0.71)	0	0.32	
Male	2	1,579	0.61 (0.35,1.07)	79	0.03	

HR, hazard ratio; CI, confidence interval; HFH, heart failure re-hospitalization.

#### 3.4.2 All-cause mortality

A pooled analysis of 6 studies (*n* = 4,715) revealed significantly lower all-cause mortality with LBBAP compared to BiVP (HR: 0.83, 95% CI: 0.71–0.96; *I*^2^ = 0%; Figure 2B). Subgroup analyses showed differential treatment effects by study design and sex. A significant reduction in mortality was observed in retrospective studies (HR: 0.80, 95% CI: 0.68–0.82) but not in prospective studies (HR: 1.02, 95% CI: 0.67–1.56). When stratified by sex, LBBAP did not demonstrate a statistically significant benefit for either males (HR: 0.93, 95% CI: 0.73–1.18) or females (HR: 1.08, 95% CI: 0.66–1.75). Leave-one-out sensitivity analysis indicated that the overall result was generally robust, as the pooled HR remained largely consistent (*I*^2^ range: 0–14.3%; HR range: 0.8–0.9). However, exclusion of the study by Morcos et al. (2025) led to a loss of statistical significance for the association (HR: 0.9, 95% CI: 0.73–1.11). Detailed forest plots and subgroup data are provided in Figure 2B and Table 3.

#### 3.4.3 Hospitalization for heart failure

A pooled analysis of 5 studies (*n* = 4,224) demonstrated a significantly lower risk of HFH with LBBAP vs. BiVP (HR: 0.58, 95% CI: 0.50–0.67; *I*^2^ = 13.9%; Figure 2C). Subgroup analysis revealed a significant treatment benefit in prospective studies (HR: 0.47, 95% CI: 0.35–0.62), retrospective studies (HR: 0.63, 95% CI: 0.53–0.75), and females (HR: 0.47, 95% CI: 0.31–0.71). In contrast, no significant benefit was observed in males (HR: 0.61, 95% CI: 0.35–1.07). Leave-one-out sensitivity analysis reaffirmed the robustness of these findings (*I*^2^ range: 0–30.7%; HR range: 0.53–0.60), with no single study materially altering the pooled estimate. Supporting data are shown in Figure 2C and Table 3.

#### 3.4.4 Echocardiographic response and super-response rates

**Figure 3:**
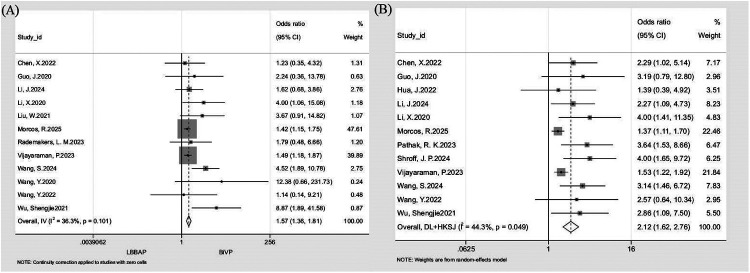
Forest plot comparing echocardiographic response and super-response rate between LBBAP and BiVP. **(A)** Echocardiographic response rate; **(B)** Super-response rate.

Separate pooled analyses of 12 studies demonstrated significantly higher echocardiographic response (*n* = 3,729; OR: 1.57, 95% CI: 1.36–1.81; *I*^2^ = 36.3%; Figure 3A) and super-response rates (*n* = 3,804; OR: 2.12, 95% CI: 1.62–2.76; *I*^2^ = 44.3%; Figure 3B) with LBBAP compared with BiVP. The benefit of LBBAP was consistent across all prespecified subgroups. For response rate, results favored LBBAP in prospective (OR: 3.13, 95% CI: 1.85–5.30), retrospective (OR: 1.51, 95% CI: 1.30–1.75), ≤1-year (OR: 3.23, 95% CI: 1.86–5.59), and >1-year (OR: 1.51, 95% CI: 1.30–1.75) follow-up groups. Similar trends were observed for super-response rate in prospective (OR: 2.95, 95% CI: 2.07–4.22), retrospective (OR: 1.51, 95% CI: 1.31–1.76), ≤1-year (OR: 2.79, 95% CI: 1.70–4.58), and >1-year (OR: 1.61, 95% CI: 1.39–1.85) subgroups. Leave-one-out sensitivity analyses confirmed result stability for both outcomes (response: *I*^2^ = 12.4–42%, OR range = 1.52–1.72; super-response: *I*^2^ = 28.3–49.1%, OR range = 1.96–2.44), with no individual study exerting a disproportionate effect (Table 4).

**Table 4:** Subgroup analysis of echocardiographic response rate and super-response rate.

Subgroup	Meta-analysis			Heterogeneity		Test for interaction *P*
	No. of studies	Total population	OR (95%Cl)	*I*^2^ (%)	*P*	
Echocardiographic response rate						
Research design						0.01
Prospective study	8	501	3.13 (1.85,5.30)	0	0.47	
Retrospective study	4	3,228	1.51 (1.30,1.75)	54	0.09	
Follow–up time						0.01
≤1 year	7	459	3.23 (1.86,5.59)	9	0.36	
>1 year	5	3,270	1.51 (1.30,1.75)	40	0.16	
Super-response rate						
Research design						0.001
Prospective study	8	576	2.95 (2.07,4.22)	0	0.90	
Retrospective study	4	3,228	1.51 (1.31,1.76)	46	0.14	
Follow-up time						0.05
≤1 year	4	292	2.79 (1.70,4.58)	0	0.87	
>1 year	8	3,512	1.61 (1.39,1.85)	52	0.04	

OR, odds ratio; CI, confidence interval.

#### 3.4.5 Improvement in LVEF

**Figure 4:**
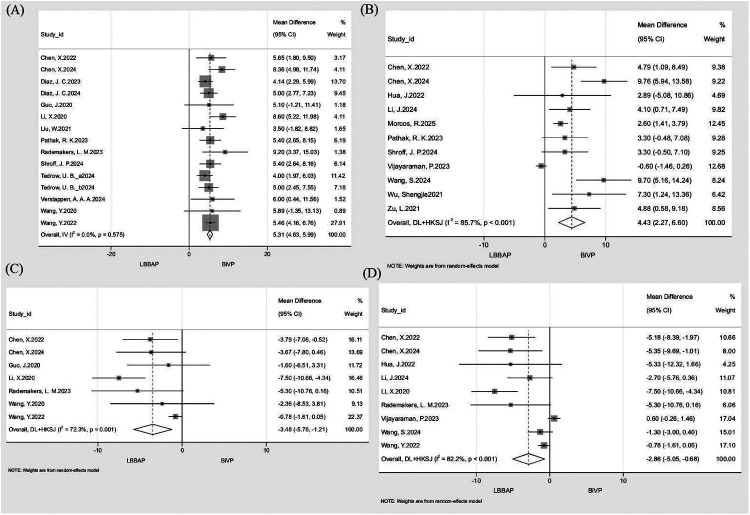
Forest plot comparing echocardiographic remodeling outcomes between LBBAP and BiVP. **(A)** △LVEF at 3-6 months; **(B)** △LVEF at ≥12 months; **(C)** △LVEDD at 3-6 months; **(D)** △LVEDD at ≥12 months.

Separate pooled analyses of 14 studies (*n* = 2,057) for 3–6-month and 11 studies (*n* = 3,775) for ≥12-month LVEF improvement revealed significantly greater LVEF gains with LBBAP vs. BiVP. The 3–6-month analysis showed a mean difference (MD) of 5.31% (95% CI: 4.63–5.99; *I*^2^ = 0%; Figure 4A), while the ≥12-month analysis yielded an MD of 4.43% (95% CI: 2.27–6.60; *I*^2^ = 85.7%; Figure 4B). Leave-one-out sensitivity analysis demonstrated consistent stability for 3–6-month outcomes (*I*^2^ = 0%; MD range = 5.17–5.50), whereas exclusion of Vijayaraman (2023) in the ≥12-month analysis reduced heterogeneity to 58.7% and increased MD to 5.03%, indicating this study as the major heterogeneity source. Meta-regression was conducted to further explore heterogeneity. Although individual covariates were not statistically significant (all *P* > 0.05), their combined inclusion explained most of the observed heterogeneity, reducing residual heterogeneity to 46.7% (Adj *R*^2^ = 70.8%). Detailed results are provided in Supplementary Table S4.

#### 3.4.6 Improvement in LVEDD

Separate pooled analyses of 7 studies (*n* = 450) for 3–6-month and 9 studies (*n* = 2,083) for ≥12-month LVEDD improvement showed significantly greater LVEDD reduction with LBBAP than with BiVP. The 3–6-month analysis demonstrated an MD of −3.48 mm (95% CI: −5.76 to −1.21; *I*^2^ = 72.3%; Figure 4C), while the ≥12-month analysis yielded an MD of −2.86 mm (95% CI: −5.05 to −0.68; *I*^2^ = 82.2%; Figure 4D). Sensitivity analysis indicated that excluding Wang et al., 2022 from the 3–6-month group reduced heterogeneity to 11.9% and shifted the MD to −4.53 mm, identifying it as the primary heterogeneity driver. In the ≥12-month group, leave-one-out analysis yielded *I*^2^ values between 74.2 and 84.4% and MD ranging from −3.64 to −2.02 mm, confirming robustness. Meta-regression for the 3–6-month and ≥12-month analyses found no single covariate to be significant (all *P* > 0.05), yet the combined model markedly reduced heterogeneity (residual *I*^2^ = 12.2%, Adj *R*^2^ = 64.5% for 3–6 months; residual *I*^2^ = 44.7%, Adj *R*^2^ = 64.3% for ≥12 months). Detailed results are provided in Supplementary Table S4.

## 4 Discussion

In this systematic review and meta-analysis, which included 24 studies comprising 6,538 participants, LBBAP demonstrated significant superiority over BiVP across multiple domains—including the composite endpoint, all-cause mortality, HFH, echocardiographic response and super-response rate, and improvements in ejection fraction (EF) and left ventricular end-diastolic diameter (LVEDD).

Our analysis demonstrated that LBBAP reduced the risk of the composite endpoint by 33% compared with BiVP, driven by a 42% lower HFH and a 17% reduction in all-cause mortality. These results are consistent with prior studies. For instance, Parlavecchio et al. conducted a meta-analysis of 10 studies involving 1,063 participants, reporting an approximately 40% lower HFH with LBBAP vs. BiVP, although mortality was not assessed (9). Similarly, a pooled analysis by Leventopoulos et al. (11 studies; *n* = 3,141) found that LBBAP was associated with a 30% reduction in all-cause mortality and a 40% lower HFH compared with BiVP (12). Methodologically, whereas those earlier analyses employed the Mantel–Haenszel method to pool outcomes as risk ratios—which for time-to-event outcomes such as all-cause mortality and HFH only reflects differences in cumulative event rates at follow-up end without adjusting for inter-study heterogeneity in follow-up duration, potentially introducing pooling bias—our study specifically used HR with 95% CI for time-to-event outcomes and exclusively incorporated adjusted HRs from each included study, thereby enhancing the reliability and methodological rigor of our findings. Furthermore, in sensitivity analysis for all-cause mortality, exclusion of the study by Morcos et al. (2025) led to loss of statistical significance for the mortality benefit of LBBAP (HR: 0.9, 95% CI: 0.73–1.11), suggesting that this study may have exerted a disproportionate influence on the pooled effect and indicating that the impact of LBBAP on all-cause mortality still requires further validation through additional high-quality studies.

Interpretation of sex-specific outcomes from our meta-analysis indicates that, based on available data, LBBAP did not demonstrate a statistically significant reduction in all-cause mortality for either males (HR: 0.93, 95% CI: 0.73–1.18) or females (HR: 1.08, 95% CI: 0.66–1.75) when compared to BiVP. It is important to note that these stratified estimates were derived from a limited subset of studies, resulting in wide confidence intervals—particularly for females—which reflects limited statistical power and precludes definitive conclusions regarding the presence or absence of a true sex-specific treatment effect. Therefore, the current evidence is insufficient to confirm or rule out mortality benefits in specific sex subgroups. Future large-scale trials with pre-specified, adequately powered sex-stratified analyses are essential to clarify potential differential responses to LBBAP.

Collectively, existing evidence consistently supports the superiority of LBBAP over BiVP in improving heart failure outcomes despite variability in study populations and follow-up durations. This advantage likely arises from multiple interrelated mechanisms. LBBAP directly restores physiological conduction through His-Purkinje system activation, avoiding BiVP-induced dyssynchrony (7), thereby optimizing transmural stress distribution, reducing myocardial oxygen consumption, and mitigating adverse remodeling (40). Additionally, LBBAP-induced QRS narrowing is associated with reduced transmural repolarization dispersion (e.g., Tpeak–Tend interval) (41, 42), which may decrease arrhythmogenic risk. Moreover, by activating the left bundle branch network, LBBAP enhances electromechanical synchrony, accelerates ventricular depolarization, and improves contraction coordination—mechanisms that may collectively reduce acute decompensation events.

Our analysis also confirmed that LBBAP yields significantly greater short-term cardiac improvements, reflected by a clinically meaningful 5.3% absolute increase in LVEF and a 3.48 mm reduction in LVEDD within 3–6 months. These findings align with prior reports by Cheng et al. (43) and Parlavecchio et al. (9), reinforcing the superior reverse remodeling potential of LBBAP. The magnitude of these changes is clinically significant. Importantly, the observed heterogeneity in short-term LVEDD reduction (*I*^2^ = 72.3%) diminished to near-homogeneity (*I*^2^ = 11.9%) after excluding the study by Wang et al. (20), which exclusively included non-ICM patients with complete LBBB. This observation underscores two potential effect modifiers of LBBAP efficacy: enhanced remodeling capacity in non-ischemic myocardial substrates and heightened electromechanical responsiveness in the presence of complete LBBB.

The superiority of LBBAP was further corroborated by significantly higher echocardiographic response rates (74.4% vs. 64.8%) and super-response rates (42.6% vs. 30.6%) relative to BiVP. These advantages persisted beyond 12 months, with sustained improvements in both LVEF and LVEDD. However, substantial heterogeneity was noted in long-term outcomes (e.g., *I*^2^ = 85.7% for ≥12-month LVEF improvement), necessitating cautious interpretation. Sensitivity analyses identified the study by Vijayaraman et al. (13) as a major source of heterogeneity, likely due to a markedly higher proportion of ICM patients in its BiVP cohort compared with the LBBAP group. This suggests that interstudy differences in baseline patient composition significantly contributed to variability. Meta-regression analysis, although limited by the small number of studies, showed that incorporating baseline variables collectively reduced heterogeneity (*I*^2^ for ≥12-month LVEF improvement decreased from 85.7% to 46.7%), indicating that these factors jointly account for much of the observed variation. Overall, these findings reinforce the physiological and clinical advantages of LBBAP, while underscoring the need for large, rigorously designed studies to delineate key effect modifiers across patient subgroups.

Our study reinforces the substantial advantages of LBBAP over BiVP for CRT; however, two major barriers hinder its widespread clinical adoption. First, long-term safety data remain insufficient. Current evidence, limited to a median follow-up of 1–3 years and none beyond 5 years, fails to elucidate the risks of late lead dysfunction or delayed septal injury—critical concerns for a lifelong pacing therapy. Second, the absence of standardized criteria for verifying “true LBB capture” results in pronounced inter-center variability in procedural success and clinical efficacy, thereby impeding consistent implementation. Accordingly, future research should prioritize large-scale randomized trials with extended follow-up (≥5 years) and coordinated multi-center efforts to establish uniform diagnostic and technical guidelines. Such initiatives are essential for the safe and systematic integration of LBBAP into routine clinical practice.

This study has several methodological limitations. First, the predominance of observational data (23 of 24 studies; only one randomized controlled trial) introduces a high risk of residual confounding. Although adjusted hazard ratios for time-to-event outcomes were pooled to mitigate bias, baseline imbalances—such as sex distribution and ischemic cardiomyopathy prevalence—may still distort treatment effect estimates. Second, the median follow-up of approximately 13 months is short relative to the lifelong course of cardiac pacing, likely underestimating long-term outcomes including all-cause mortality and device-related complications (e.g., lead dysfunction, septal injury) and limiting robust comparison of the two pacing modalities' durability. Third, most studies failed to clearly differentiate LBBP from LVSP within the broader LBBAP category, obscuring potential differences in physiological and clinical effects. Fourth, the absence of patient-level data stratified by modifiers such as heart-failure etiology, conduction-block subtype, and baseline QRS duration precluded the identification of subgroups most likely to benefit from LBBAP. Finally, outcome measures such as echocardiographic response and LVEF improvement may be affected by publication bias; while sensitivity analyses supported the robustness of results, treatment benefits might still be overestimated.

## 5 Conclusions

This systematic review and meta-analysis demonstrate that LBBAP significantly reduces the composite clinical endpoint, all-cause mortality, and heart-failure hospitalization, while enhancing cardiac remodeling compared with BiVP in patients undergoing CRT. Large, multicenter randomized controlled trials are warranted to confirm long-term efficacy and clarify sex-specific differences in response, thereby advancing precision and equity in CRT delivery.

## Data Availability

The original contributions presented in the study are included in the article/Supplementary Material, further inquiries can be directed to the corresponding author.
