# Supplementary material for: Left bundle branch area pacing vs. biventricular pacing significantly improves clinical outcomes and cardiac remodeling in cardiac resynchronization therapy: a systematic review and meta-analysis
Source: Front Cardiovasc Med. 2025 Nov 21;12:1644033. doi: 10.3389/fcvm.2025.1644033 (PMC12678356; doi:10.3389/fcvm.2025.1644033)
Supplement: Supplementary file 1 [file Table1.docx]

| **Search Strategies（Search Date: 2025.05.03）** | | |
| --- | --- | --- |
| **Pubmed** |  |  |
| #1 | "Cardiac Resynchronization Therapy"[Mesh]  OR "Cardiac Pacing, Artificial"[Mesh]  OR "biventricular pacing"[All Fields] OR BiVP[All Fields] OR CRT[All Fields]  OR "left bundle branch area pacing"[All Fields] OR LBBAP[All Fields]  OR "left bundle branch pacing"[All Fields] OR LBBP[All Fields]  OR "His bundle pacing"[All Fields] OR HBP[All Fields] | 52366 |
| #2 | "Heart Failure"[MeSH Terms] OR "Cardiac Failure"[All Fields] OR "Heart Decompensation"[All Fields] OR "decompensation heart"[All Fields] OR "Congestive Heart Failure"[All Fields] OR "heart failure congestive"[All Fields] OR "heart failure right sided"[All Fields] OR "right sided heart failure"[All Fields] OR "heart failure right sided"[All Fields] OR "right sided heart failure"[All Fields] OR "heart failure left sided"[All Fields] OR "left sided heart failure"[All Fields] OR "heart failure left sided"[All Fields] OR "left sided heart failure"[All Fields] OR "Myocardial Failure"[All Fields] OR "Heart Failure"[All Fields] OR "Cardiac Failure"[All Fields] OR "Congestive Heart Failure"[All Fields] OR "right sided heart failure"[All Fields] OR "right sided heart failure"[All Fields] OR "left sided heart failure"[All Fields] OR "left sided heart failure"[All Fields] | 302132 |
| #3 | (("Echocardiography"[Mesh] OR ( ("left ventricular ejection fraction" OR LVEF OR "LV ejection fraction" OR "LV EF" OR EF) OR ("left ventricular end-systolic volume" OR LVESV OR "LV end-systolic volume") OR ("left ventricular end-diastolic volume" OR LVEDV OR "LV end-diastolic volume") OR ("electromechanical synchrony" OR "mechanical synchrony") OR ("Tissue Doppler Imaging" OR "Doppler Tissue Imaging" OR TDI) OR ("strain imaging" OR "myocardial strain") OR ("3D echocardiography" OR "three-dimensional echocardiography") )) OR ( "Mortality"[Mesh] OR "Death"[Mesh] OR ( "death"[All Fields] OR "mortality"[All Fields] OR "fatal outcome"[All Fields] OR "death rate"[All Fields] OR "cardiac death"[All Fields] OR "heart failure death"[All Fields] ) ) OR ( "Hospitalization"[Mesh] OR ( "hospitalization"[All Fields] OR "hospital readmission"[All Fields] OR "readmission rate"[All Fields] OR "rehospitalization"[All Fields] OR "rehospitalization rate"[All Fields] ) )) | 3031160 |
| #4 | （"Meta-Analysis"[Publication Type] OR "Review"[Publication Type] OR "Systematic Review"[Publication Type] OR "Case Reports"[Publication Type] OR "Editorial"[Publication Type] OR "Letter"[Publication Type] OR "News"[Publication Type] OR "Autobiography"[Publication Type] OR "Biography"[Publication Type] OR "Comment"[Publication Type] OR "Retracted Publication"[Publication Type] OR "Historical Article"[Publication Type] OR "Patient Education Handout"[Publication Type] OR "Practice Guideline"[Publication Type] OR "Expression of Concern"[Publication Type] OR "Dataset"[Publication Type] OR "Technical Report"[Publication Type] OR "Webcast"[Publication Type] OR "Video-Audio Media"[Publication Type] OR "Preprint"[Publication Type] OR "Collected Work"[Publication Type] OR "Clinical Trial Protocol"[Publication Type] OR ("Animals"[Mesh] NOT "Humans"[Mesh] )） |  |
| #5 | #1 and #2 and #3 not #4 | 3897 |
| **embase** |  |  |
| #1 | ((cardiac resynchronization therapy/exp) OR (cardiac pacing, artificial/exp) OR ("biventricular pacing" OR BiVP OR CRT) OR ("left bundle branch area pacing" OR LBBAP) OR ("left bundle branch pacing" OR LBBP) OR ("His bundle pacing" OR HBP)) | 93424 |
| #2 | ('heart failure'/exp) OR "Cardiac Failure" OR "Heart Decompensation" OR "decompensation heart" OR "Congestive Heart Failure" OR "heart failure congestive" OR "heart failure right sided" OR "right sided heart failure" OR "heart failure left sided" OR "left sided heart failure" OR "Myocardial Failure" OR "Heart Failure" | 833669 |
| #3 | echocardiography'/exp OR 'left ventricular ejection fraction' OR lvef OR 'lv ejection fraction' OR 'lv ef' OR ef OR 'left ventricular end - systolic volume' OR lvesv OR 'lv end - systolic volume' OR 'left ventricular end - diastolic volume' OR lvedv OR 'lv end - diastolic volume' OR 'electromechanical synchrony' OR 'mechanical synchrony' OR 'tissue doppler imaging' OR 'doppler tissue imaging' OR tdi OR 'strain imaging' OR 'myocardial strain' OR '3d echocardiography' OR 'three - dimensional echocardiography' OR 'mortality'/exp OR 'death'/exp OR 'death' OR 'mortality' OR 'fatal outcome' OR 'death rate' OR 'cardiac death' OR 'heart failure death' OR 'hospitalization'/exp OR 'hospitalization' OR 'hospital readmission' OR 'readmission rate' OR 'rehospitalization' OR 'rehospitalization rate' | 4712366 |
| #4 | randomized controlled trial'/exp OR 'clinical trial'/exp OR 'experimental studies'/exp OR 'crossover studies' OR 'field trials' OR 'observational studies'/exp OR 'cohort studies'/exp OR 'case-control studies'/exp OR 'cross-sectional studies'/exp OR 'case series'/exp OR 'descriptive studies' OR 'longitudinal studies'/exp OR 'validation studies'/exp OR 'feasibility studies'/exp OR 'methodological studies'/exp | 4791678 |
| #5 | ("Meta - Analysis"/it OR "Review"/it OR "Systematic Review"/it OR "Case Reports"/it OR "Editorial"/it OR "Letter"/it OR "News"/it OR "Autobiography"/it OR "Biography"/it OR "Comment"/it OR "Retracted Publication"/it OR "Historical Article"/it OR "Patient Education Handout"/it OR "Practice Guideline"/it OR "Expression of Concern"/it OR "Dataset"/it OR "Technical Report"/it OR "Webcast"/it OR "Video - Audio Media"/it OR "Preprint"/it OR "Collected Work"/it OR "Clinical Trial Protocol"/it) OR (("Animals"/exp) NOT ("Humans"/exp)) | 11836561 |
| #6 | (#1 and #2 and #3 and #4) not #5 | 4629 |
| **cochrane** |  |  |
| #1 | MeSH descriptor: [Cardiac Resynchronization Therapy] explode all trees | 609 |
| #2 | MeSH descriptor: [Cardiac Pacing, Artificial] explode all trees | 1911 |
| #3 | biventricular pacing OR BiVP OR CRT OR left bundle branch area pacing OR LBBAP OR left bundle branch pacing OR LBBP OR His bundle pacing OR HBP | 7146 |
| #4 | #1 OR #2 OR #3 | 8198 |
| #5 | MeSH descriptor: [Heart Failure] explode all trees | 14786 |
| #6 | ("Cardiac Failure" OR "Heart Decompensation" OR "Myocardial Failure" OR "Congestive Heart Failure" OR "heart failure congestive" OR "right sided heart failure" OR "heart failure right sided" OR "left sided heart failure" OR "heart failure left sided") | 9220 |
| #7 | #5 OR #6 | 20926 |
| #8 | MeSH descriptor: [Echocardiography] explode all trees | 5228 |
| #9 | ("left ventricular ejection fraction" OR LVEF OR "LV ejection fraction" OR "LV EF" OR EF OR "left ventricular end-systolic volume" OR LVESV OR "LV end-systolic volume" OR "left ventricular end-diastolic volume" OR LVEDV OR "LV end-diastolic volume" OR "electromechanical synchrony" OR "mechanical synchrony" OR "Tissue Doppler Imaging" OR "Doppler Tissue Imaging" OR TDI OR "strain imaging" OR "myocardial strain" OR "3D echocardiography" OR "three-dimensional echocardiography") | 23478 |
| #10 | MeSH descriptor: [Mortality] explode all trees | 18501 |
| #11 | MeSH descriptor: [Death] explode all trees | 3544 |
| #12 | ("death" OR "mortality" OR "fatal outcome" OR "death rate" OR "cardiac death" OR "heart failure death") | 185115 |
| #13 | MeSH descriptor: [Hospitalization] explode all trees | 20450 |
| #14 | ("hospitalization" OR "hospital readmission" OR "readmission rate" OR "rehospitalization" OR "rehospitalization rate") | 66978 |
| #15 | #8 or #9 or #10 or #11 or #12 or #13 or #14 | 254143 |
| #16 | ("Conference proceeding" OR "Trial registry record" OR "Book or thesis" OR "Erratum" OR "Expression of concern" OR "Retraction of publication" OR "Retracted publication"):pt | 836842 |
| #17 | MeSH descriptor: [Animals] explode all trees | 892603 |
| #18 | MeSH descriptor: [Humans] explode all trees | 889322 |
| #19 | #17 Not #18 | 3281 |
| #20 | #16 or #19 | 840121 |
| #21 | #4 and #7 and #15 not #20 | 693 |
| **CNKI** |  |  |
| #1 | (SU % "心脏再同步治疗" OR SU % "人工心脏起搏" OR SU % "双心室起搏" OR SU % "BiVP" OR SU % "CRT" OR SU % "左束支区域起搏" OR SU % "LBBAP" OR SU % "左束支起搏" OR SU % "LBBP" OR SU % "希氏束起搏" OR SU % "HBP") OR (KY % "心脏再同步治疗" OR KY % "人工心脏起搏" OR KY % "双心室起搏" OR KY % "BiVP" OR KY % "CRT" OR KY % "左束支区域起搏" OR KY % "LBBAP" OR KY % "左束支起搏" OR KY % "LBBP" OR KY % "希氏束起搏" OR KY % "HBP") OR (AB % "心脏再同步治疗" OR AB % "人工心脏起搏" OR AB % "双心室起搏" OR AB % "BiVP" OR AB % "CRT" OR AB % "左束支区域起搏" OR AB % "LBBAP" OR AB % "左束支起搏" OR AB % "LBBP" OR AB % "希氏束起搏" OR AB % "HBP") | 26801 |
| #2 | (SU % "心力衰竭" OR SU % "心脏衰竭" OR SU % "心脏失代偿" OR SU % "失代偿性心脏" OR SU % "充血性心力衰竭" OR SU % "右侧心力衰竭" OR SU % "左侧心力衰竭" OR SU % "心肌衰竭") OR (KY % "心力衰竭" OR KY % "心脏衰竭" OR KY % "心脏失代偿" OR KY % "失代偿性心脏" OR KY % "充血性心力衰竭" OR KY % "右侧心力衰竭" OR KY % "左侧心力衰竭" OR KY % "心肌衰竭") OR (AB % "心力衰竭" OR AB % "心脏衰竭" OR AB % "心脏失代偿" OR AB % "失代偿性心脏" OR AB % "充血性心力衰竭" OR AB % "右侧心力衰竭" OR AB % "左侧心力衰竭" OR AB % "心肌衰竭") | 167640 |
| #3 | (SU % "超声心动图" OR SU % "左心室射血分数" OR SU % "LVEF" OR SU % "LV射血分数" OR SU % "LV EF" OR SU % "EF" OR SU % "左心室收缩末期容积" OR SU % "LVESV" OR SU % "左室收缩末期容积" OR SU % "左心室舒张末期容积" OR SU % "LVEDV" OR SU % "左室舒张末期容积" OR SU % "机电同步性" OR SU % "机械同步性" OR SU % "组织多普勒成像" OR SU % "多普勒组织成像" OR SU % "TDI" OR SU % "应变成像" OR SU % "心肌应变" OR SU % "三维超声心动图") OR (SU % "死亡率" OR SU % "死亡" OR SU % "致命结局" OR SU % "死亡率" OR SU % "心脏性死亡" OR SU % "心力衰竭死亡") OR (SU % "住院" OR SU % "再住院" OR SU % "再入院率" OR SU % "重新住院" OR SU % "重新住院率") OR (KY % "超声心动图" OR KY % "左心室射血分数" OR KY % "LVEF" OR KY % "LV射血分数" OR KY % "LV EF" OR KY % "EF" OR KY % "左心室收缩末期容积" OR KY % "LVESV" OR KY % "LV收缩末期容积" OR KY % "左心室舒张末期容积" OR KY % "LVEDV" OR KY % "LV舒张末期容积" OR KY % "机电同步性" OR KY % "机械同步性" OR KY % "组织多普勒成像" OR KY % "多普勒组织成像" OR KY % "TDI" OR KY % "应变成像" OR KY % "心肌应变" OR KY % "三维超声心动图") OR (KY % "死亡率" OR KY % "死亡" OR KY % "致命结局" OR KY % "死亡率" OR KY % "心脏性死亡" OR KY % "心力衰竭死亡") OR (KY % "住院" OR KY % "再住院" OR KY % "再入院率" OR KY % "重新住院" OR KY % "重新住院率") OR (AB % "超声心动图" OR AB % "左心室射血分数" OR AB % "LVEF" OR AB % "LV射血分数" OR AB % "LV EF" OR AB % "EF" OR AB % "左心室收缩末期容积" OR AB % "LVESV" OR AB % "LV收缩末期容积" OR AB % "左心室舒张末期容积" OR AB % "LVEDV" OR AB % "LV舒张末期容积" OR AB % "机电同步性" OR AB % "机械同步性" OR AB % "组织多普勒成像" OR AB % "多普勒组织成像" OR AB % "TDI" OR AB % "应变成像" OR AB % "心肌应变" OR AB % "三维超声心动图") OR (AB % "死亡率" OR AB % "死亡" OR AB % "致命结局" OR AB % "死亡率" OR AB % "心脏性死亡" OR AB % "心力衰竭死亡") OR (AB % "住院" OR AB % "再住院" OR AB % "再入院率" OR AB % "重新住院" OR AB % "重新住院率") | 2210434 |
| #4 | (SU % "随机对照试验" OR SU % "临床试验" OR SU % "实验研究" OR SU % "交叉研究" OR SU % "现场试验" OR SU % "观察性研究" OR SU % "队列研究" OR SU % "病例对照研究" OR SU % "横断面研究" OR SU % "病例系列" OR SU % "描述性研究" OR SU % "纵向研究" OR SU % "验证性研究" OR SU % "可行性研究" OR SU % "方法学研究") OR (KY % "随机对照试验" OR KY % "临床试验" OR KY % "实验研究" OR KY % "交叉研究" OR KY % "现场试验" OR KY % "观察性研究" OR KY % "队列研究" OR KY % "病例对照研究" OR KY % "横断面研究" OR KY % "病例系列" OR KY % "描述性研究" OR KY % "纵向研究" OR KY % "验证性研究" OR KY % "可行性研究" OR KY % "方法学研究") OR (AB % "随机对照试验" OR AB % "临床试验" OR AB % "实验研究" OR AB % "交叉研究" OR AB % "现场试验" OR AB % "观察性研究" OR AB % "队列研究" OR AB % "病例对照研究" OR AB % "横断面研究" OR AB % "病例系列" OR AB % "描述性研究" OR AB % "纵向研究" OR AB % "验证性研究" OR AB % "可行性研究" OR AB % "方法学研究") | 2445257 |
| #5 | #1 and #2 and #3 and #4 | 346 |
