# Supplementary material for: Left bundle branch area pacing vs. biventricular pacing significantly improves clinical outcomes and cardiac remodeling in cardiac resynchronization therapy: a systematic review and meta-analysis
Source: Front Cardiovasc Med. 2025 Nov 21;12:1644033. doi: 10.3389/fcvm.2025.1644033 (PMC12678356; doi:10.3389/fcvm.2025.1644033)
Supplement: Supplementary file 3 [file Table3.docx]

| Supplementary Table 3. Assessment of Publication Bias and Adjustment Using Trim-and-Fill Analysis for Various Outcomes.​ | | | | | |
| --- | --- | --- | --- | --- | --- |
| **Outcome Indicator** | **Effect Measure** | **Test for Dichotomous Outcomes （Harbord Test Pvalue）** | **Test for Continuous Outcomes （Egger's Test Pvalue）** | **No. of Imputed Studies** | **Adjusted Result after Trim-and-Fill** |
| Composite endpoint | HR | 0.005 | NR | 2 | 0.69 (0.62,0.78) |
| All-cause mortality | HR | 0.345 | NR | 0 | NR |
| HFH | HR | 0.038 | NR | 2 | 0.61 (0.53,0.72) |
| Echocardiographic response | OR | 0.006 | NR | 5 | 1.48 (0.96,2.29) |
| Echocardiographic super-response rates | OR | < 0.001 | NR | 6 | 1.6 (1.16,2.19) |
| ΔLVEF at 3-6 months | MD, % | NR | 0.193 | 1 | 5.37 (4.64,6.11) |
| ΔLVEF at ≥12 months | MD, % | NR | 0.005 | 0 | NR |
| ΔLVEDD at 3-6 months | MD, mm | NR | 0.071 | 0 | NR |
| ΔLVEDD at ≥12 months | MD, mm | NR | 0.006 | 3 | ‘-2.03(-3.92,-0.13) |
| Abbreviations: HR, Hazard Ratio; NR, Not Reported; OR, Odds Ratio; MD, Mean Difference; HFH, Heart Failure Re-hospitalization; ΔLVEF, Change in Left Ventricular Ejection Fraction; ΔLVEDD, Change in Left Ventricular End-Diastolic Diameter. | | | | | |
