# Supplementary material for: Left bundle branch area pacing vs. biventricular pacing significantly improves clinical outcomes and cardiac remodeling in cardiac resynchronization therapy: a systematic review and meta-analysis
Source: Front Cardiovasc Med. 2025 Nov 21;12:1644033. doi: 10.3389/fcvm.2025.1644033 (PMC12678356; doi:10.3389/fcvm.2025.1644033)
Supplement: Supplementary file 4 [file Table4.docx]

| Supplementary Table 4. Meta - Regression Analysis of Factors Associated with Changes in Left Ventricular Function Indices. | | | | | | |
| --- | --- | --- | --- | --- | --- | --- |
| Variable | Coefficient | Standard Error | P-value | 95% CI | I²ₙₛ | Adj R² |
| ΔLVEF at ≥12 months |  |  |  |  |  |  |
| Δfemale ratio | -11.46 | 39.71 | 0.792 | (-137.84, 114.93) | 46.7% | 70.8% |
| ΔNICM ratio | 12.13 | 23.34 | 0.639 | (-62.14, 86.39) |  |  |
| ΔLBBB ratio | 33.19 | 34.06 | 0.402 | (-75.21, 141.59) |  |  |
| ΔQRS duration | -0.07 | 0.88 | 0.941 | (-2.87, 2.73) |  |  |
| Follow up | -0.24 | 0.31 | 0.496 | (-1.22, 0.75) |  |  |
| Constant | 10.88 | 12.23 | 0.439 | (-28.04, 49.79) |  |  |
| ΔLVEDD at 3-6 months |  |  |  |  |  |  |
| Δfemale ratio | -8.3 | 12.15 | 0.618 | (-162.62, 146.02) | 12.2% | 64.5% |
| ΔNICM ratio | 21.23 | 27.57 | 0.582 | (-329.12, 371.57) |  |  |
| ΔQRS duration | -0.7 | 0.53 | 0.415 | (-7.44, 6.04) |  |  |
| Follow up | 0.11 | 0.21 | 0.695 | (-2.57, 2.79) |  |  |
| Constant | 0.79 | 3.52 | 0.86 | (-43.97, 45.54) |  |  |
| ΔLVEDD at ≥12 months | |  |  |  |  |  |
| Δfemale ratio | 16.14 | 22.19 | 0.543 | (-79.33, 111.61) | 44.7% | 64.3% |
| ΔNICM ratio | 15.05 | 29.13 | 0.657 | (-110.28, 140.38) |  |  |
| ΔQRS duration | -0.22 | 0.33 | 0.583 | (-1.65, 1.22) |  |  |
| Follow up | 0.16 | 0.27 | 0.607 | (-0.99, 1.31) |  |  |
| Constant | -6.51 | 8.03 | 0.503 | (-41.05, 28.03) |  |  |
| Abbreviations: CI, Confidence Interval; I^2^ns, Residual Heterogeneity; AdjR^2^, Adjusted Coefficient of Determination; ΔLVEF, Change in Left Ventricular Ejection Fraction; ΔLVEDD, Change in Left Ventricular End-Diastolic Diameter; NICM, Non-Ischemic Cardiomyopathy; LBBB, Left Bundle Branch Block. | | | | | | |
