# Supplementary material for: Left bundle branch area pacing vs. biventricular pacing significantly improves clinical outcomes and cardiac remodeling in cardiac resynchronization therapy: a systematic review and meta-analysis
Source: Front Cardiovasc Med. 2025 Nov 21;12:1644033. doi: 10.3389/fcvm.2025.1644033 (PMC12678356; doi:10.3389/fcvm.2025.1644033)
Supplement: Supplementary file 5 [file Image1.pdf]

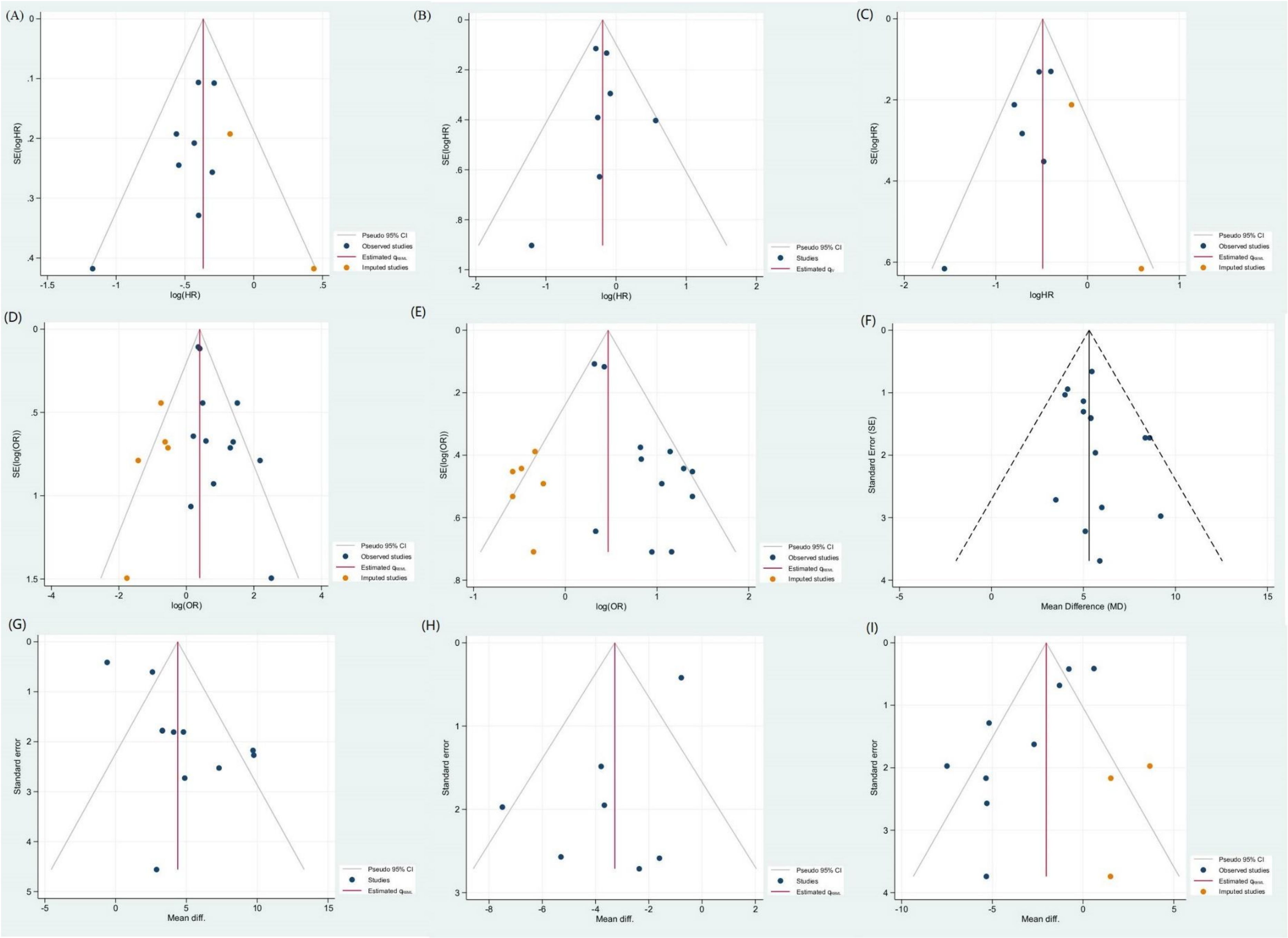

(A)
SE(logHR)
log(HR)
Pseudo 95% CI
Observed studies
Estimated q
Imputed studies
(B)
SE(logHR)
log(HR)
Pseudo 95% CI
Studies
Estimated q
(C)
SE(logHR)
logHR
Pseudo 95% CI
Observed studies
Estimated q
Imputed studies
(D)
SE(log(OR))
log(OR)
Pseudo 95% CI
Observed studies
Estimated q
Imputed studies
(E)
SE(log(OR))
log(OR)
Pseudo 95% CI
Observed studies
Estimated q
Imputed studies
(F)
Standard Error (SE)
Mean Difference (MD)
(G)
Standard error
Mean diff.
Pseudo 95% CI
Studies
Estimated q
(H)
Standard error
Mean diff.
Pseudo 95% CI
Studies
Estimated q
(I)
Standard error
Mean diff.
Pseudo 95% CI
Observed studies
Estimated q
Imputed studies

Supplementary Figure 1. Funnel Plots with Trim-and-Fill Analysis for LBBAP vs. BiVP.

Funnel plots display the original studies (blue circles) and the imputed studies (orange circles) from the trim-and-fill analysis. The solid and dashed lines represent the original and adjusted effect estimates, respectively. (A) Composite endpoint (all-cause mortality/HFH); (B) All-cause mortality; (C) HFH; (D) Echocardiographic response rate; (E) Super-response rate; (F) △LVEF at 3-6 months; (G) △LVEF at ≥12 months; (H) △LVEDD at 3-6 months; (I) △LVEDD at ≥12 months. BiVP, biventricular pacing; HFH, heart failure hospitalization; HR, hazard ratio; LBBAP, left bundle branch area pacing; LVEDD, left ventricular end-diastolic diameter; LVEF, left ventricular ejection fraction; OR, odds ratio.
